# Growth of *Escherichia coli* in Minimal Media Supplemented with *N*^6^-Methylated but Not *N*^6^,*N*^6^-Dimethylated Purines Is Supported by Adenosine Deaminase Add

**DOI:** 10.3390/biom16060758

**Published:** 2026-05-22

**Authors:** Jaunius Urbonavičius, Augusta Ivaškė, Daiva Tauraitė

**Affiliations:** Department of Chemistry and Bioengineering, Vilnius Gediminas Technical University, Saulėtekio Avenue 11, 10223 Vilnius, Lithuania; jaunius.urbonavicius@vilniustech.lt (J.U.); augusta.ivaske@vilniustech.lt (A.I.)

**Keywords:** *Escherichia coli*, modified heterocyclic base, adenine/guanine auxotrophy, methylated purines, metabolism, adenine/adenosine deaminase, AdeC, Add

## Abstract

*N*^6^-methyladenine and *N*^6^,*N*^6^-dimethyladenine are the heterocyclic bases present in the RNA of eukaryotic and bacterial cells and play important regulatory roles. How the degradation of such modified nucleic acids, and the subsequent demethylation of modified heterocyclic bases, occurs in the bacterium *Escherichia coli* is not established. Here, we investigated the growth of adenine auxotroph strains in a minimal M9 medium supplemented with either *N*^6^-methyladenine or *N*^6^,*N*^6^-dimethyladenine. We found that *N*^6^-methyladenine supported the growth of *∆purH*::Km but not that of the *∆purA*::Km strain, whereas *N*^6^,*N*^6^-dimethyladenine did not support the growth of either adenine auxotroph. Similar experiments performed using structurally related 2-amino-*N*^6^-methylpurine and 2-amino-*N*^6^,*N*^6^-dimethylpurine bases—using *∆guaA*::Km, *∆guaB*::Km, and *∆purH*::Km guanine auxotrophs—demonstrated that growth of only the *∆guaB*::Km mutant was supported by 2-amino-*N*^6^-methylpurine but not by its dimethylated counterpart. We expressed and purified C-teminus 6xHis tagged *E. coli* adenine/adenosine deaminases AdeC and Add and tested their substrate specificity. We demonstrated that AdeC protein does not catalyse deamination of either *N*^6^-methyl- or *N*^6^,*N*^6^-dimethyladenine, whereas Add catalyses deamination of *N*^6^-methyl- but not that of *N*^6^,*N*^6^-dimethyladenosine. Based on our findings, biochemical pathways leading to the demodification and return into metabolism of *N*^6^-methyladenine and 2-amino-*N*^6^-methylpurine in *E. coli* are proposed.

## 1. Introduction

RNA is a multifaceted molecule that participates in numerous cellular functions. To perform those functions properly, RNA molecules are decorated by different chemical groups. They are mostly found in tRNA, with more than 170 different species present [1]. These modifications are crucial for tRNA folding, structure, and function [2,3]. They are important for translation [4] and maintaining protein homeostasis; their loss leads to the development of neurological diseases, like intellectual disability, multiple sclerosis, schizophrenia, Alzheimer’s disease [5,6] and cancer [7]. In addition to tRNA, a variety of modified nucleosides are present in different RNA species, such as mRNA, rRNA, snRNA, snoRNA, miRNAs, and lncRNAs, where they play an important role in gene expression [8,9,10]. Chemical modifications are also used for new types of mRNA vaccines, like those directed against various types of cancer [11,12]. Although knowledge on the formation of RNA modifications and their cellular role is well established, much less is known about RNA degradation and the return to metabolism of the corresponding nucleotides, nucleosides, and heterocyclic bases. Studying such enzymes might be of importance for their potential use in chemoenzymatic drug synthesis [13].

One way to investigate the degradation of modified RNA and the subsequent fate of corresponding heterocyclic bases is the use of *E. coli* or *Saccharomyces cerevisiae* auxotrophy-based systems. The modified heterocyclic bases are used as sources of natural heterocyclic bases (e.g., uracil) in corresponding minimal media. If the gene whose product converts a particular heterocyclic base into an unmodified one does not exist in the host cells and growth is, therefore, not observed, then that gene is looked for via environmental metagenomic libraries. In this way, a gene that encodes the TudS protein that catalyses the conversion of 2-thiouracil to uracil was discovered [14]. However, some modified pyrimidine bases such as *N*^4^-methylcytosine were shown to support the growth of uracil auxotrophs, attesting to the presence of the corresponding demodification enzymes in *E. coli*. The biochemical pathway for the conversion of *N*^4^-methylcytosine to uracil was proposed [15]. Similarly, several other modified pyrimidines were tested for support of the growth of *S. cerevisiae* uracil auxotrophs. *N*^4^-acetylcytosine was shown to support the growth of such strains in minimal media. However, when some other modified bases (such as 3-methyluracil) were used, very slow growth of uracil auxotrophs was observed [16]. In summary, *N*^4^-methylcytosine and *N*^4^-acetylcytosine are readily converted to uracil by *E. coli* and/or *S. cerevisiae*, whereas 2-thiouracil is not. These observations indicate the presence or absence of the corresponding demodification enzymes in the microorganisms tested.

Approaches similar to those applied to investigate the metabolism of the modified pyrimidines could be used for the modified purine bases. Such compounds could be used together with the corresponding adenine or guanine auxotrophs to test whether they support the growth of the corresponding *E. coli* or *S. cerevisiae* mutant. The purine whose catabolism is best studied is caffeine (1,3,7-methylxantine), widely used due to its stimulant effect in coffee, tea, and cacao [17]. Caffeine degradation occurs via *N*-demethylation or C-8 oxidation pathways [18]. Numerous bacterial strains associated with caffeine degradation have been isolated and the presence of the corresponding degradation enzymes in their genome has been demonstrated, but none of such bacteria belong to the *Escherichia* genus [19]. This feature was used to investigate caffeine metabolism in *E. coli*, where the Δ*guaB* mutant that needs xanthine for growth in minimal media was used. The combination of different ones provided on the complementing plasmid N-demethylation (*ndm*) genes and various caffeine derivatives (caffeine itself, theobromine or theophylline) leads to the appearance of xanthine in the growth medium and the growth of the *E. coli* Δ*guaB* strain [20]. We took a similar approach to test the growth of several *E. coli* purine biosynthesis mutants in minimal media where different modified purine bases were used as the source of purines. We found that *N*^6^-methyladenine or 2-amino-*N*^6^-methylpurine but not their respective dimethylated counterparts supports the growth of such *E. coli* strains. We proposed the biochemical pathways for such conversion and, to support our model, expressed, purified, and tested the enzymatic activities of the corresponding deaminases. The experiments that describe our findings are presented below.

## 2. Materials and Methods

### 2.1. Synthesis of N^6^-Methylated Adenines and 2-Amino-N^6^-methylated Purines

Chemicals, solvents, and kits were purchased from Sigma-Aldrich (Merck group, Darmstadt, Germany) and Thermo Fisher Scientific Baltics (Vilnius, Lithuania) and used without further purification. *N*^6^-methyladenine, *N*^6^,*N*^6^-dimethyladenine, 2-amino-*N*^6^-methylpurine and 2-amino-*N*^6^,*N*^6^-dimethylpurine were synthesized by modifying synthesis method reported in the literature [21]. Thin-layer chromatography (TLC) was performed on aluminium sheets coated with silica gel 60 F_254_ (Merck, Darmstadt, Germany). Flash column chromatography was performed on silica gel 60 (0.063–0.200 mm) (Merck, Darmstadt, Germany) using chloroform/methanol mixtures 10/1 → 10/2 as mobile phase. NMR spectra were recorded in DMSO-d_6_ on a Bruker Ascend 400 ^1^H NMR—400 MHz and ^13^C NMR—101 MHz (Bruker BioSpin, Rheinstetten, Germany). Chemical shifts were reported in ppm relative to the solvent resonance signal as an internal standard. UV spectra were measured using a SPECORD 210 UV/Vis spectrophotometer (Analytik Jena, Jena, Germany). The HPLC analyses were performed using ultra-fast liquid chromatography system (UFLC LC-20AD) with a photodiode array detector (SPD-M20A, Shimadzu, Kyoto, Japan). Chromatographic separation was conducted using a Waters Symmetry300 C18, 3.5 µm HPLC column, 2.1 mm × 150 mm (Waters corporation, Milford, MA, USA) at 40 °C and a mobile phase consisting of a 0.01% formic acid/water solution (solvent A) and a 0.01% formic acid/methanol solution (solvent B).

General Procedure

A mixture of 0.65 mmol of 6-chloropurine or 2-amino-6-chloropurine and 3.9 mmol of CH_3_NH_2_ or (CH_3_)_2_NH solution in 40% in water was dissolved in ethanol (5 mL). The mixture was refluxed for 6–8 h. The completion of reaction was checked by TLC (CHCl_3_/CH_3_OH, 9/1). When the reaction was completed, ethanol and water were removed under reduced pressure and the residue was purified by flash column chromatography using silica gel sorbent and chloroform/methanol mixtures, 10/1 → 10/2 as an eluent. The following compounds were prepared using this general procedure:

***N*^6^-Methyladenine**: Yield 94 mg (97%) white solid, R_f_ = 0.46 (CHCl_3_/CH_3_OH, 9/1). UV (CH_3_OH) λ_max_ (ε) 267 (15.4 × 10^3^) nm (M^−1^ × cm^−1^), purity 99% (HPLC). ^1^H NMR (400 MHz, DMSO-d_6_) δ 12.89 (s, 1H, NH), 8.20 (s, 1H, CH=), 8.08 (s, 1H, CH=), 7.59 (s, 1H, NHCH_3_), 2.95 (s, 3H, CH_3_). ^13^C NMR (DMSO-d_6_, 101 MHz): δ = 155.00, 152.89, 152.29, 138.87, 119.56, 28.29.

***N*^6^,*N*^6^-Dimethyladenine**: Yield 100 mg (94%), white solid. R_f_ = 0.59 (CHCl_3_/CH_3_OH, 9/1). UV (CH_3_OH) λ_max_ (ε) 275 (17.1 × 10^3^) nm (M^−1^ × cm^−1^), purity 98% (HPLC). ^1^H NMR (400 MHz, DMSO-d_6_) δ 12.98 (s, 1H, NH), 8.18 (s, 1H, CH=), 8.09 (s, 1H, CH=), 3.36 (s, 6H, CH_3_). ^13^C NMR (DMSO-d_6_, 101 MHz): δ = 154.67, 152.26, 151.51, 138.25, 119.40, 40.59.

**2-Amino-*N*^6^-methylpurine**: Yield 104 mg (97%), white solid. R_f_ = 0.31 (CHCl_3_/CH_3_OH, 9/1). UV (CH_3_OH) λ_max_ (ε) 278 (10.0 × 10^3^) nm (M^−1^ × cm^−1^), purity 99% (HPLC). ^1^H NMR (400 MHz, DMSO-d_6_) δ 12.70 (s, 1H, NH), 8.44–7.89 (m, 1H, NHCH_3_), 7.84 (s, 1H, CH=), 6.61 (s, 2H, NH_2_) 2.94 (s, 3H, CH_3_). ^13^C NMR (DMSO-d_6_, 101 MHz): δ = 158.31, 154.36, 150.03, 137.83, 120.18, 27.63.

**2-Amino-*N*^6^,*N*^6^-dimethylpurine**: Yield 110 mg (95%), white foam. R_f_ = 0.50 (CHCl_3_/CH_3_OH, 9/1). UV (CH_3_OH) λ_max_ (ε) 284 (11.4 × 10^3^) nm (M^−1^ × cm^−1^), purity 99% (HPLC). ^1^H NMR (400 MHz, DMSO-d_6_) δ 12.06 (s, 1H, NH), 7.66 (s, 1H, CH=), 5.70 (s, 2H, NH_2_), 3.17 (s, 6H, CH_3_). ^13^C NMR (DMSO-d_6_, 101 MHz): δ = 159.85, 155.04, 153.87, 134.97, 113.81, 49.06.

### 2.2. Bacterial Strains and Growth Media

The respective adenine and/or guanine auxotrophs, *∆purA*::K_m_, *∆purH*::K_m_, *∆guaA*::K_m_, and *∆guaB*::K_m_ in the genetic background of *E. coli* K-12 BW25113 were obtained from the Keio collection through Horizon Discovery (Cambridge, UK). M9 medium containing necessary salts (final concentration is: KH_2_PO_4_, 3 g/L, NaCl, 0.5 g/L, Na_2_HPO_4_, 6.8 g/L, NH_4_Cl, 1 g/L), 0.4% of glucose, and 15 µg/mL of kanamycin, was supplemented with 20 µg/mL of either adenine, *N*^6^-methyladenine, *N*^6^,*N*^6^-dimethyladenine, guanine, 2-amino-*N*^6^-methylpurine, 2-amino-*N*^6^,*N*^6^-dimethylpurine to test bacterial growth. For growing bacteria in solid media, 2% agar was used. Bacterial strains were inoculated by streaking onto solid M9 minimal media with different modified adenines or guanines and respective controls (adenine or guanine for positive control or no purines added for negative control) and incubated at 37 °C for up to 7 days to monitor the bacterial growth.

To remove the K_m_ cassette, the 708-FLPe plasmid that contains FLP-recombinase and chloramphenicol resistance marker (Gene Bridges GmbH, Heidelberg, Germany) was introduced to *∆purH*::K_m_ strain by standard chemical transformation. The loss of K_m_ resistance cassette and the plasmid was achieved by growing this strain in LB medium at 30 °C and changing the temperature to 37 °C according to the manufacturer’s recommendations. The loss of resistance markers was confirmed by streaking the obtained strains on LB plates with 15 µg/mL kanamycin or 15 µg/mL chloramphenicol (Figure S1). The growth of such constructed strain in M9 minimal medium was tested as described above.

### 2.3. Determination of the Growth Rate of E. coli Guanine and Adenine Auxotrophs

The *E. coli ∆purH*::K_m_ strain was grown in M9 minimal medium supplemented with adenin/e and kanamycin at 37 °C overnight. The cells were washed with 0.9% NaCl solution twice and transferred to new M9 minimal medium supplemented with 15 µg/mL kanamycin and 200 µg/mL of either adenine, *N*^6^-methyladenine, or *N*^6^,*N*^6^-dimethyladenine. The cells were aerobically grown at 37 °C. Samples for measuring the optical density at 600 nm wavelength were taken each hour. The generation time g was calculated according to the following formula

g = t × ln2/(lnX_t_ − lnX_0_) (1)

where t is the time in hours, X_t_ is OD_600_ at time t, and X_0_ is OD_600_ at time 0.

The *E. coli ∆guaB*::K_m_ strain was grown in M9 minimal medium supplemented with guanine and kanamycin at 37 °C overnight. The cells were washed with 0.9% NaCl solution twice and transferred to new M9 minimal medium supplemented with 15 µg/mL kanamycin and 200 µg/mL of either guanine, 2-amino-*N*^6^-methylpurine, or 2-amino-*N*^6^,*N*^6^-dimethylpurine. The growth rate of these bacterial strains was determined according to the procedure described above.

### 2.4. Assessment of the of Heterocyclic Base and Nucleoside Accumulation in Growth Media

The *∆purA*::K_m_ strain was grown in M9 minimal medium supplemented with 100 µg/mL of adenine and 15 µg/mL of kanamycin at 37 °C overnight. The cells were then transferred to the new M9 minimal medium supplemented with kanamycin and 100 µg/mL adenine and grown for 6 h. Subsequently, the cells were washed with a 0.9% NaCl solution thrice and transferred into new M9 minimal medium supplemented with kanamycin and 100 µg/mL of either *N*^6^-methyladenine, *N*^6^,*N*^6^-dimethyladenine, *N*^6^-methyladenosine, or *N*^6^,*N*^6^-dimethyladenosine. Cells were subsequently incubated at 37 °C overnight.

Samples for HPLC analysis were taken after 18 h of incubation of cell culture with methylated adenines/adenosines. A total of 600 µL of sample was centrifuged and 0.4 mL of methanol was added to 0.4 mL of the obtained supernatant to kill the remaining cells and precipitate the salts. The samples were centrifuged again, and 10 μL of the respective solution was loaded onto a C18, 4.6 mm × 150 mm column. HPLC analysis was performed as described above in Section 2.1.

### 2.5. Purification of E. coli AdeC and Add Proteins and Determination of Their Enzymatic Activity

AdeC protein containing the 6xHis tag at C-terminus was purified using the pET-21a(+)-adeC plasmid, purchased from GenScript Biotech (Nanjing, China). The plasmid was transformed into the *E. coli* Rosetta (DE3) strain (Merck KGaA, Darmstadt, Germany). The resulting bacteria were grown in LB medium with 100 µg/mL of ampicillin at 37 °C until OD_600_ reached around 0.8; the isopropyl β-D-thiogalactopyranoside (IPTG) was added to the final concentration of 0.5 mM along with 1 mM MnCl_2_, and the culture was grown for another 3 h. Cells were collected by centrifugation and resuspended in 20 mM Tris-HCl, pH 8.0, with 0.3 M NaCl. The cells were then disrupted using B-PER™ Bacterial Protein Extraction Reagent (ThermoFisher Scientific Baltics, Vilnius, Lithuania). Cell debris was removed by centrifugation at 12,000× *g* for 30 min. The resulting supernatant was loaded onto a Ni-NTA column (Cytiva Sweden AB, Uppsala, Sweden), previously equilibrated with 20 mM Tris-HCl, pH 8.0, with 0.3 M NaCl. The proteins were applied to the column and washed with 5 column volumes of the equilibration buffer. The adsorbed proteins were eluted with 20 mM Tris-HCl, pH 8.0, with 0.3 M NaCl using a linear gradient of 10–500 mM imidazole. Protein-containing fractions were pooled and desalted against 20 mM Tris-HCl, pH 7.5 using the 10K and 30K Amicon concentrators (Merck KGaA, Darmstadt, Germany). The purity of recombinant AdeC was confirmed by SDS-PAGE electrophoresis. The protein concentration was measured using the Bradford method.

Enzymatic reactions were carried out at 37 °C in 20 mM Tris-HCl, pH 7.5 for 1 h. A 40 µL final reaction mixture contained 2.5 µg of purified protein and 25 mM of the corresponding methylated adenine as substrate. After incubation, 1 µL of the reaction mixture was used for thin-layer chromatography analysis that was performed on aluminium sheets coated with silica gel 60 F_254_ using a chloroform/methanol mixture (5:1) as a mobile phase. The spots were visualized under 254 nm UV light.

For HPLC analysis, 60 µL of methanol was added to the incubation mixture, centrifuged, and 1 µL was loaded onto a Waters Symmetry300 C18, 3.5 µm HPLC column as described above.

The Add protein was purified similarly to AdeC, except that pET-21a(+)-add plasmid was used for expression, and 0.1 M ZnSO_4_ was added before induction. The enzymatic reactions with Add protein were performed as for the AdeC protein, but methylated adenosines were used as substrates.

## 3. Results and Discussion

### 3.1. Assessment of Adenine and Guanine Auxotroph Growth in M9 Solid Media

**Figure 1:**
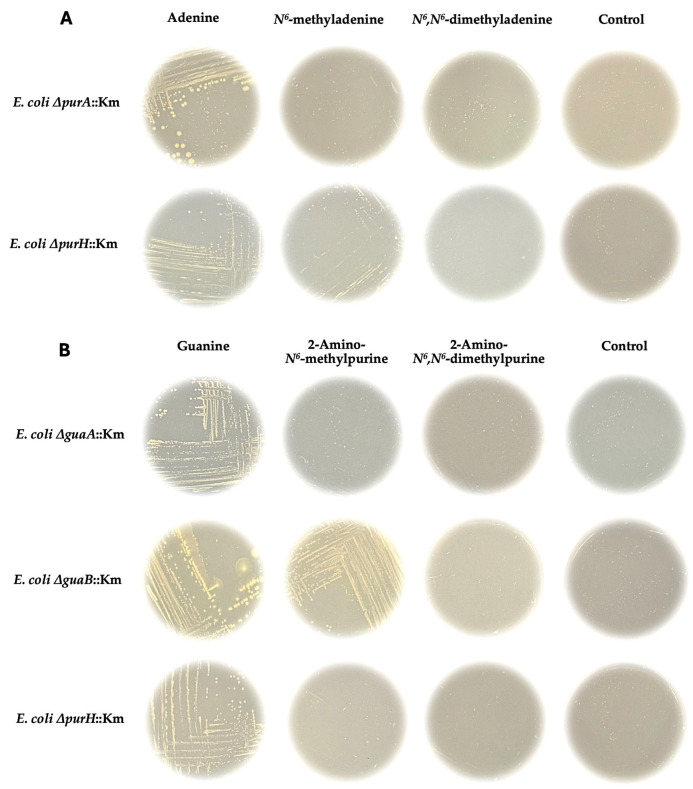
Growth of *E. coli* adenine and guanine auxotrophs in M9 minimal agar medium supplemented with different heterocyclic bases. (**A**) *∆purA*::K_m_ and *∆purH*::K_m_ strains grown in medium supplemented with adenine, *N*^6^-methyladenine or *N*^6^,*N*^6^-dimethyladenine. (**B**) *∆guaA*::K_m_; *∆guaB*::K_m_, and *∆purH*::K_m_ strains grown in medium supplemented with guanine, 2-amino-*N*^6^-methylpurine or 2-amino-*N*^6^,*N*^6^-dimethylpurine. M9 minimal agar medium without any heterocyclic bases was used as control. The plates were incubated at 37 °C for 7 days.

As an initial approach, several natural and synthetic modified heterocyclic purine bases were tested for their ability to support the growth of respective adenine or guanine auxotrophs. In the case of modified adenine heterocyclic bases, respective *E. coli ∆purA*::K_m_ and *∆purH*::K_m_ adenine auxotrophs were used. *N*^6^-methyladenine was demonstrated to support the growth of the *∆purH*::K_m_ but not that of *∆purA*::K_m_ strain in M9 minimal medium. In contrast, *N*^6^,*N*^6^-dimethyladenine did not promote growth or either strain in this medium (Figure 1A). Similarly, *∆guaA*::K_m_, *∆guaB*::K_m_, and *∆purH*::K_m_ strains were grown in M9 minimal medium supplemented with either 2-amino-*N*^6^-methylpurine or 2-amino-*N*^6^,*N*^6^-dimethylpurine. In this case, it was demonstrated that 2-amino-*N*^6^-methylpurine supports the growth of *∆guaB*::K_m_ but not that of *∆guaA*::K_m_ or *∆purH*::K_m_ mutant (Figure 1B). Since the *purH* gene is the first gene of the bicistronic operon together with *purD*, insertion of the K_m_ cassette as in *∆purH*::K_m_ strain could cause polar effects affecting the growth of *E. coli*. Therefore, the minimal media growth experiments were performed with *∆purH* strain, in which the kanamycin resistance gene was removed. We did not observe any difference in growth phenotypes compared to *∆purH*::K_m_ strains, attesting that the presence of the antibiotic resistance marker does not affect the growth (Figure S1).

### 3.2. Determination of E. coli Auxotroph Growth Rates in Liquid M9 Minimal Medium

**Figure 2:**
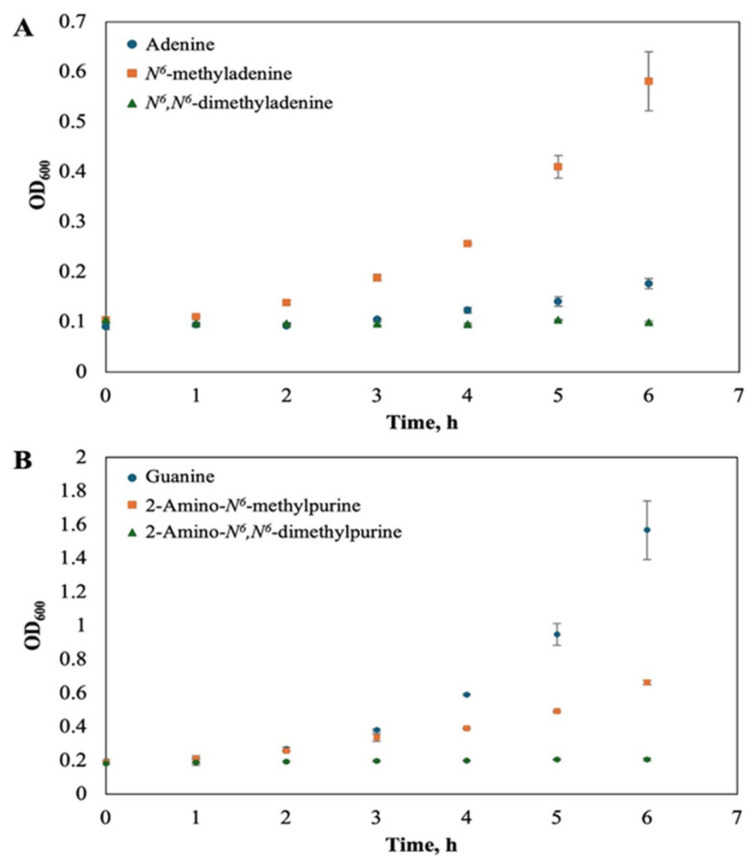
Growth of the *∆guaB*::K_m_ and *∆purH*::K_m_ strains in liquid M9 minimal medium supplemented with different heterocyclic bases. (**A**) growth of *∆purH*::K_m_ in medium supplemented with adenine, *N*^6^-methyladenine or *N*^6^,*N*^6^-dimethyladenine. (**B**) growth of *∆guaB*::K_m_ in medium supplemented with guanine, 2-amino-*N*^6^-methylpurine or 2-amino-*N*^6^,*N*^6^-dimethylpurine. Error bars show the standard deviation of the three experiments.

For better evaluation of the growth rate of *∆purH*::K_m_ and *∆guaB*::K_m_ strains, M9 minimal medium supplemented with various modified heterocyclic bases was used. The *∆purH*::K_m_ strain was grown overnight in liquid M9 minimal medium supplemented with adenine, then harvested, washed with 0.9% NaCl twice and transferred into fresh M9 medium supplemented with either adenine, *N*^6^-methyladenine or *N*^6^,*N*^6^-dimethyladenine. Surprisingly, very good growth was observed in the medium with added *N*^6^-methyladenine, compared to that with adenine or *N*^6^,*N*^6^-dimethyladenine (Figure 2A). A generation time for this compound was about 2 h, whereas in the presence of adenine, the generation time observed was about 5.6 h (Table 1). In contrary, very little if any growth was observed in the medium supplemented with *N*^6^,*N*^6^-dimethyladenine, with a generation time of about 170 h.

In the case of the *∆guaB*::K_m_ strain, it was grown overnight in liquid M9 medium supplemented with guanine. The cells were then harvested, washed with 0.9% NaCl twice, and transferred into fresh medium supplemented with either guanine, 2-amino-*N*^6^-methylpurine or 2-amino-*N*^6^,*N*^6^-dimethylpurine. In this case, cells were growing faster in the medium supplemented with guanine than that supplemented with *N*^6^-methyguanine, whereas very little if any growth was observed when 2-amino-*N*^6^,*N*^6^-dimethylpurine was used as a substrate (Figure 2B). The generation time of the *∆guaB*::K_m_ strain in M9 medium with guanine was about 1.7 h, whereas in the presence of 2-amino-*N*^6^-methylpurine, it was around 3 h. However, when 2-amino-*N*^6^,*N*^6^-dimethylpurine was used as a substrate, the generation time was around 49 h (Table 1).

### 3.3. Biochemical Pathways Leading to the Conversion of Modified Heterocyclic Bases to Their Unmodified Counterparts

**Figure 3:**
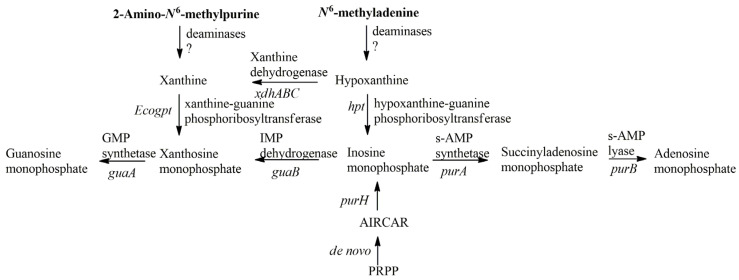
Purine nucleotide synthesis including the proposed mechanism of intermediate formation using *N*^6^-methyladenine and 2-amino-*N*^6^-methylpurine as substrates.

The observed growth phenotypes for different mutants in different M9 media led us to investigate the biochemical pathways that would lead to conversion of modified heterocyclic bases into unmodified ones, thus supporting growth. For this purpose, we used previous knowledge on de novo synthesis of purines [22,23]. It is well established that purine nucleotide biosynthesis starts with 5-phosphoribosyl-1-pyrophosphate (PRPP) and proceeds in 10 steps until IMP (inosine 5′-monophosphate) is formed (Figure 3). Then, IMP turns into XMP (xanthosine 5′-monophosphate, catalysed by the GuaB protein) and subsequently into GMP (guanosine 5′-monophosphate, catalysed by the GuaA protein). IMP is also converted into s-AMP (succinyl-adenosine 5′-monophosphate, catalysed by the PurA protein) and subsequently into AMP (adenosine 5′-monophosphate, catalysed by the PurB protein).

The obtained purine monophosphates may be further used for the synthesis of the nucleic acids. Our observation that the growth of *∆purH*::K_m_ but not of *∆purA*::K_m_ mutant is supported by *N*^6^-methyladenine suggests that the transformation of this compound proceeds via the formation of hypoxanthine that is further converted into IMP. Consequently, in the *∆purH*::K_m_ mutant, it may be converted into both GMP and AMP, leading to the growth phenotype. The reason why *N*^6^-methyladenine is the preferred growth substrate over adenine could be that the formation of both GMP and AMP through the IMP intermediate in the cell is faster than the conversion of AMP into GMP required in the absence of the de novo pathway in the *∆purH*::K_m_ mutant. However, in the *∆purA*::K_m_ mutant, IMP is not converted into s-AMP; therefore, AMP is not formed, and growth is not observed (Figure 1).

Indeed, when *∆purA*::K_m_ mutant was pregrown in medium with adenine and then transferred into that supplemented with *N*^6^-methyladenine, the accumulation of hypoxanthine was observed. In contrast, no accumulation of hypoxanthine was observed when such supplementation was performed using *N*^6^,*N*^6^-dimethyladenine (Figure S2A), attesting that this compound is not a substrate for in vivo deamination. Similar results were obtained when methylated adenosines were used instead of modified adenines under the same experimental conditions. In that case, *∆purA*::K_m_ mutant was pregrown in medium with adenine and then transferred into that, supplemented with *N*^6^-methyladenosine. Then, the accumulation of inosine and hypoxanthine was observed in the growth medium. However, when *N*^6^,*N*^6^-dimethyladenosine was used, no such conversion was observed (Figure S2B).

*N*^6^-methyladenine deamination (called then 6-methylaminopurine) was observed for both *S. typhi* and *E. coli* many years ago [24,25]. In the latter work, it was suggested that such a reaction occurs after conversion of the free base to the ribonucleoside, which then undergoes deamination to yield inosine and methylamine, possibly catabolized by adenosine deaminase. To test experimentally possible *N*^6^-methyl(adenine/adenosine) deamination, we produced in *E. coli* and purified to near homogeneity C-terminal 6xHis-tagged versions of the adenine deaminase AdeC and adenosine deaminase Add. Using such proteins, the enzymatic activity of purified proteins with respective mono- and dimethylated adenine/adenosine was tested.

### 3.4. Purification and Testing of the Enzymatic Activity of E. coli Adenine/Adenosine Deaminases AdeC and Add

**Figure 4:**
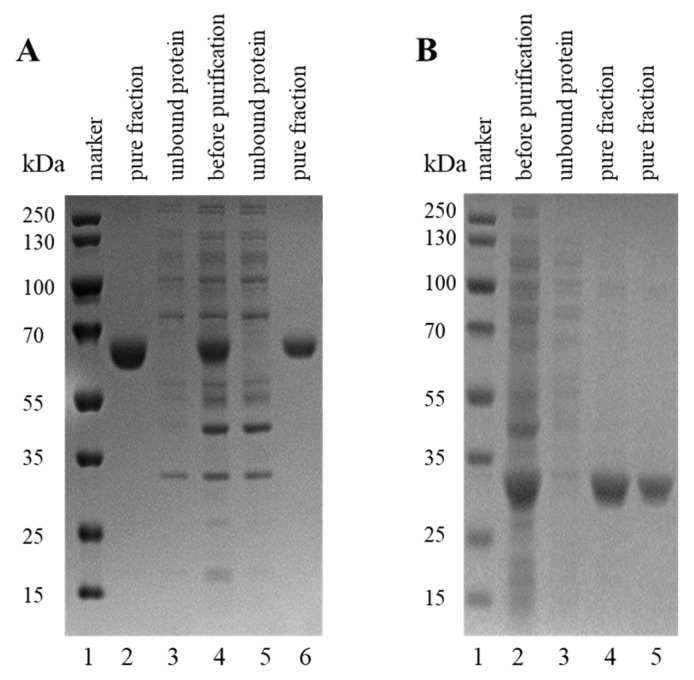
SDS-PAGE analysis of purified recombinant AdeC (**A**) and Add (**B**) proteins. Twelve % acrylamide gel was used and stained with Coomassie blue. (**A**) Lane 1—molecular weight marker. Lanes 2 and 6—fractions eluted with 250 mM imidazole in 20 mM Tris-HCl, 0.3 M NaCl, pH 8.0 buffer. Lanes 3 and 5—unbound protein fractions. Lane 4—S-12 supernatant protein fraction before purification. (**B**) Lane 1—molecular weight marker. Lane 2—S-12 supernatant protein fraction before purification. Lane 3—unbound protein fraction. Lanes 4 and 5—fractions eluted with 350 mM imidazole in 20 mM Tris-HCl, 0.3 M NaCl, pH 8.0 buffer.

For the purification of AdeC protein, pET-21a(+)-adeC plasmid was transformed into the *E. coli* Rosetta (DE3) strain. The resulting construct was grown in LB medium with ampicillin, and AdeC synthesis was induced by IPTG. Cells were collected via centrifugation, resuspended in TRIS-HCl buffer, and disrupted using B-PER™ Bacterial Protein Extraction Reagent. Cell debris was removed via centrifugation, and the resulting supernatant was used for AdeC purification by Ni ion affinity chromatography. It yielded >99% pure AdeC protein (Figure 4A), whose concentration was measured using the Bradford method. The purification of Add protein was performed similarly using the pET-21a(+)-add plasmid. It also yielded >99% pure Add protein (Figure 4B).

**Figure 5:**
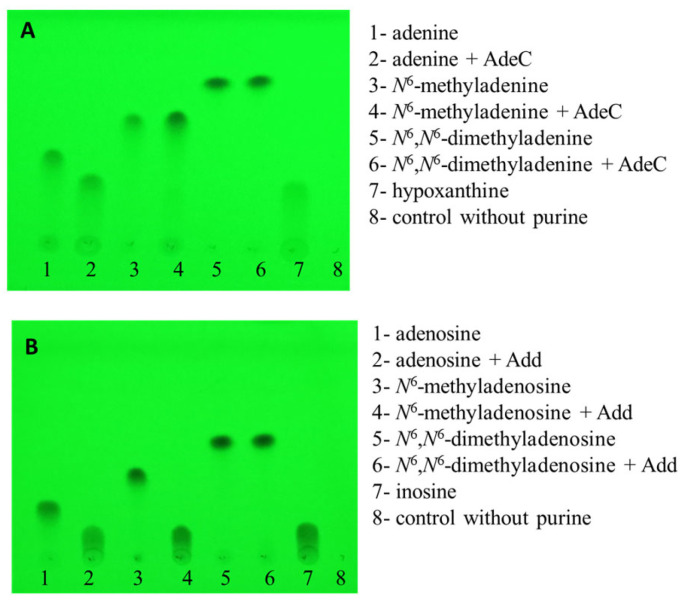
Thin-layer chromatography (TLC) of modified adenines/adenosines enzymatic reactions with AdeC (**A**) and Add (**B**) proteins.

**Figure 6:**
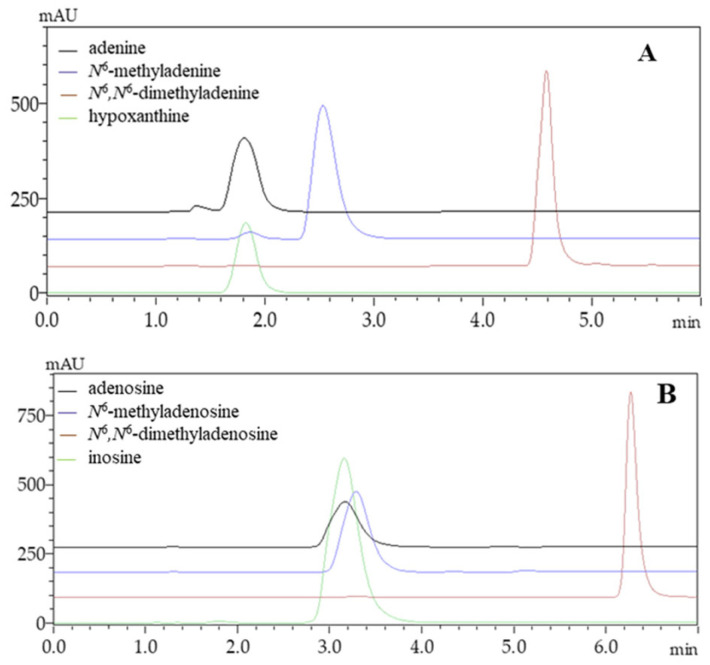
HPLC chromatograms showing the enzymatic activity of the AdeC (**A**) and Add (**B**) proteins with adenine/adenosine, their *N*^6^-methylated or *N*^6^,*N*^6^-dimethylated derivatives. Hypoxanthine and inosine are added as references.

To test the enzymatic activity of the AdeC protein, it was incubated with, respectively, adenine, *N*^6^-methyladenine, or *N*^6^,*N*^6^-dimethyladenine, and the formation of the reaction products was investigated. Using TLC analysis, it was demonstrated that only adenine was deaminated to hypoxanthine, whereas methylated adenines were not substrates of this enzyme (Figure 5A). Similar results were obtained when HPLC was used for the analysis of the reaction mixture. Only the conversion of adenine to hypoxanthine was demonstrated (Figure 6A). In conclusion, our results demonstrate that neither *N*^6^-methyladenine nor *N*^6^,*N*^6^-dimethyladenine is a substrate of the AdeC.

Next, *N*^6^-methyladenosine and *N*^6^,*N*^6^-dimethyladenosine were tested along with adenosine as substrates of adenosine deaminase Add. When this protein was incubated with such substrates, conversion of both adenosine and *N*^6^-methyladenosine but not *N*^6^,*N*^6^-dimethyladenosine to inosine was demonstrated by TLC analysis (Figure 5B). The same results were obtained when the reaction mixtures were analysed via HPLC (Figure 6B).

**Figure 7:**
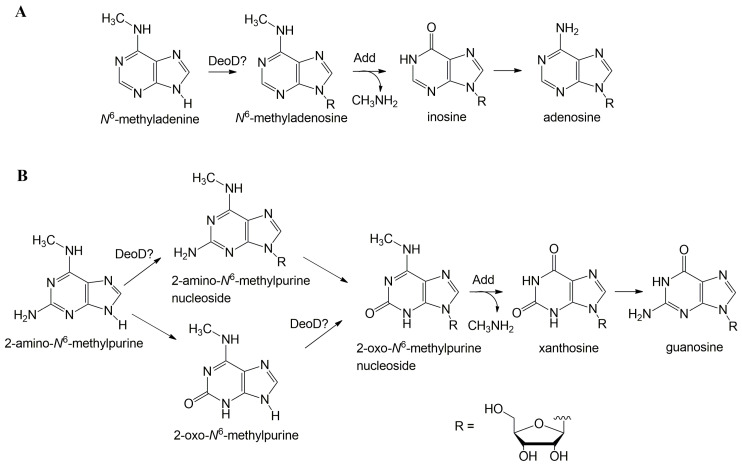
Proposed enzymatic pathways for the conversion of *N*^6^-methylated purines. (**A**) Conversion of *N*^6^-methyladenine to adenosine. (**B**) Conversion of 2-amino-*N*^6^-methylpurine to guanosine.

Our results demonstrate that the deamination of *N*^6^-methylated but not *N*^6^,*N*^6^-dimethylated purine bases occurs at the nucleoside level. The conversion of the respective bases into the nucleosides is likely performed by the purine nucleoside phosphorylase DeoD [26], which is also capable of performing the reverse reaction. Previously, the AdeC protein was cloned, expressed in *E. coli*, purified, and characterised. Of the adenine, adenosine, and AMP tested as substrates, only adenine was deaminated [27], but no methylated derivatives were used as substrates. Also, almost-50-year-old work on purified-to-about-90% adenosine deaminase from *E. coli* demonstrated that adenosine, 2-deoxyadenosine, and 6-methylaminopurine ribonucleoside (*N*^6^-methyladenosine) serve as substrates, but no kinetic values was provided for the latter compound [28]. We predict that removal of methylamine does not occur directly on 2-amino-*N*^6^-methylpurine or corresponding nucleoside since this would lead to the formation of guanine/guanosine that would support the growth of the *∆guaA*::K_m_ mutant, which is not the case. Therefore, the following enzymatic pathways for the conversion of *N*^6^-methylated purines are proposed (Figure 7). In the case of *N*^6^-methyladenine, it is first converted to *N*^6^-methyladenosine and then deaminated to inosine and subsequently converted to adenosine (Figure 7A). In case of 2-amino-*N*^6^-methylpurine, it may be first deaminated to 2-oxo-*N*^6^-methylpurine and then converted to 2-oxo-*N*^6^-methylpurine nucleoside, or 2-amino-*N*^6^-methylpurine nucleoside is formed first and then deaminated to 2-oxo-*N*^6^-methylpurine nucleoside. The methylamine is then removed to form the xanthosine and subsequently the guanosine (Figure 7B).

It is somewhat surprising that neither *N*^6^-methyladenine nor *N*^6^,*N*^6^-dimethyladenine are substrates of AdeC deaminase, since two enzymes of *Bacillus subtilis* (Bh0637 and Bsu06560) that perform a deamination reaction were discovered and characterised [29]. Also, both *N*^6^-methyladenine and *N*^6^,*N*^6^-dimethyladenine were substrates of cytokinin deaminase Patl2390 of *Pseudoalteromonas atlantica* T6c, with comparable k_cat_/K_m_ values 1.3 × 10^6^ vs. 8 × 10^6^ M^−1^ s^−1^. However, adenine and adenosine were not substrates for this enzyme [30]. Patl2390 belongs to cog1816 of Clusters of Orthologous Genes [31], and Cytoscape analysis places it in Group 3 of cog1816, whereas *E. coli* adenosine deaminase Add (*E. coli* NP_416140.1 protein) belongs to Group 5 of this COG [30]. In addition, Bh0637 and Bsu06560 *N*^6^-methyladenine deaminases belong to subgroup 2 of cog1001, while deaminases such as *E. coli* AdeC (NP_418121.1 deaminase) belong to subgroups 1 and 4 [29]. In the latter work, *N*^6^-methyladenine was demonstrated to be a good substrate for Bh0637 and Bsu06560, with k_cat_/K_m_ values of 2.9 × 10^6^ vs. 2.5 × 10^6^ M^−1^ s^−1^. As in the case of Patl2390, adenine was not a good substrate for either enzyme. *N*^6^,*N*^6^-dimethyladenine and 2-amino-*N*^6^-methylpurine were not tested as substrates, while 2,6-diamino purine was tested but was a bad substrate; therefore, no kinetic constants were calculated. Some years ago, it was demonstrated that Bsu06560 enzyme prefers *N*^6^-methyladenosine over *N*^6^-methyladenine as a substrate and established the critical amino acids responsible for this phenomenon [32]. In addition, the role of the C-terminus of this protein (also called YerA) in catalysis was recently established [33].

As described above, differences in substrate specificity between AdeC, Bsu06560, and Patl2390 may be structural due to the different COG groups or subgroups. *E. coli* AdeC protein was purified and reconstituted, and it was demonstrated that *N*^6^-methyladenine is neither a substrate nor inhibitor of this enzyme. Based on the 3D structure of *Agrobacterium tumefaciens* adenine deaminase (Atu4426), the docking of adenine to this protein and the changes in the corresponding important amino acids of *E. coli* adenine deaminase, the adenine deamination model was proposed in [34]. The presence of the *N*^6^-methyl group of adenine is likely to provide the steric hindrances for the formation of reaction intermediates and the release of the methylamine as a reaction by-product.

The structural reasons may also explain why *N*^6^,*N*^6^-dimethyladenine/*N*^6^,*N*^6^-dimethyladenosine or 2-amino-*N*^6^,*N*^6^-dimethylpurine (and likely the corresponding nucleoside) are not substrates for the demethylation reaction of AdeC/Add proteins. At least two *N*-dimethylated derivatives of heterocyclic purine bases (m^6^_2_A and m^2^_2_G) are present in RNA [1]. It is known that *E. coli* α-ketoglutarate-dependent dioxygenase AlkB and its mammalian homologs ALKBH remove alkyl adducts from nucleobases of DNA and RNA by oxidative dealkylation [35]. Human ALKBH5 has been shown to demethylate m^6^_2_A using rRNA or oligoribonucleotides as substrates, the first demethylation step being rate limiting, while m^6^A demethylation proceeds quickly [36]. Previously, we also observed that *N*^4^,*N*^4^-dimethylcytosine does not support the growth of the *E. coli pyrF* mutant that renders uracil auxotrophy in minimal medium, whereas *N*^4^-methylcytosine does [15]. Demethylation of *N*^4^,*N*^4^-dimethylcytosine may occur in a similar manner, where the first step may be limiting.

*N*^6^,*N*^6^-dimethyladenine is found in spores of the bacterium *Streptomyces alboniger* [37] and is used as a cell cycle inhibitor or inducer of apoptosis and also for oocyte activation in eukaryotic cloning experiments [38]. Its aminonucleoside derivative is the antibiotic puromycin, also isolated from *Streptomyces alboniger* and used in cell biology and biotechnology [39]. Therefore, investigations on the metabolism of *N*^6^-methylated purine bases and the corresponding nucleosides are of biomedical importance. Further bioinformatic and biochemical investigations will reveal the structural basis on the activity and substrate specificity of *E. coli* AdeC and Add adenine/adenosine deaminases.

## 4. Conclusions

*E. coli* is capable of using both *N*^6^-methyladenine and 2-amino-*N*^6^-methylpurine but not their dimethylated counterparts to support cell growth in minimal media. The removal of the methylamine groups from *N*^6^-methyladenine in vitro occurs at the nucleoside level and is mediated by adenosine deaminase Add. Further bioinformatic and biochemical analysis would reveal the reasons why the removal of the methylamine group of such purines occurs at the nucleoside level, whereas the dimethylated derivatives are not substrates of this enzyme.

## Figures and Tables

**Table 1:** Generation times of the *E. coli* guanine and adenine auxotrophs in liquid M9 minimal medium supplemented with different heterocyclic bases. Values represent the average of 3 experiments and the standard deviation.

	Adenine	*N*^6^-Methyladenine	*N*^6^,*N*^6^-Dimethyladenine
*E. coli ∆purH*::K_m_	5.58 ± 0.67 h	2.10 ± 0.10 h	170.39 ± 3.27 h
	Guanine	2-Amino-*N*^6^-methylpurine	2-Amino-*N*^6^,*N*^6^-dimethylpurine
*E. coli ∆guaB*::K_m_	1.68 ± 0.06 h	3.03 ± 0.09 h	48.73 ± 20.77 h

## Data Availability

The original contributions presented in this study are included in the article; further inquiries can be directed to the corresponding author.

## Supplementary Materials

The following supporting information can be downloaded at: https://www.mdpi.com/article/10.3390/biom16060758/s1, Figure S1: Growth of the *E. coli* *∆purH* strain in the LB with antibiotics or M9 minimal media supplemented with different heterocyclic bases; Figure S2: Accumulation of heterocyclic bases and nucleosides in media supplemented with *N*^6^-methylated adenines or adenosines.
