# Supplementary material for: Growth of Escherichia coli in Minimal Media Supplemented with N6-Methylated but Not N6,N6-Dimethylated Purines Is Supported by Adenosine Deaminase Add
Source: Biomolecules. 2026 May 22;16(6):758. doi: 10.3390/biom16060758 (PMC13296781; doi:10.3390/biom16060758)
Supplement: Supplementary file 1 [file biomolecules-16-00758-s001.zip › biomolecules-4227535-supplementary.pdf]

SUPPORTING INFORMATION

Growth of *Escherichia coli* in minimal media supplemented with N^6-methylated but not N^6,N^6-dimethylated purines is supported by adenosine deaminase Add

Jaunius Urbonavičius, Augusta Ivaškė and Daiva Tauraitė *

Department of Chemistry and Bioengineering, Vilnius Gediminas Technical University, Saulėtekio Av. 11, 10223 Vilnius, Lithuania; jaunius.urbonavicius@vilniustech.lt; augusta.ivaske@vilniustech.lt

*Correspondence: daiva.tauraite@vilniustech.lt; Tel.: +37052744839

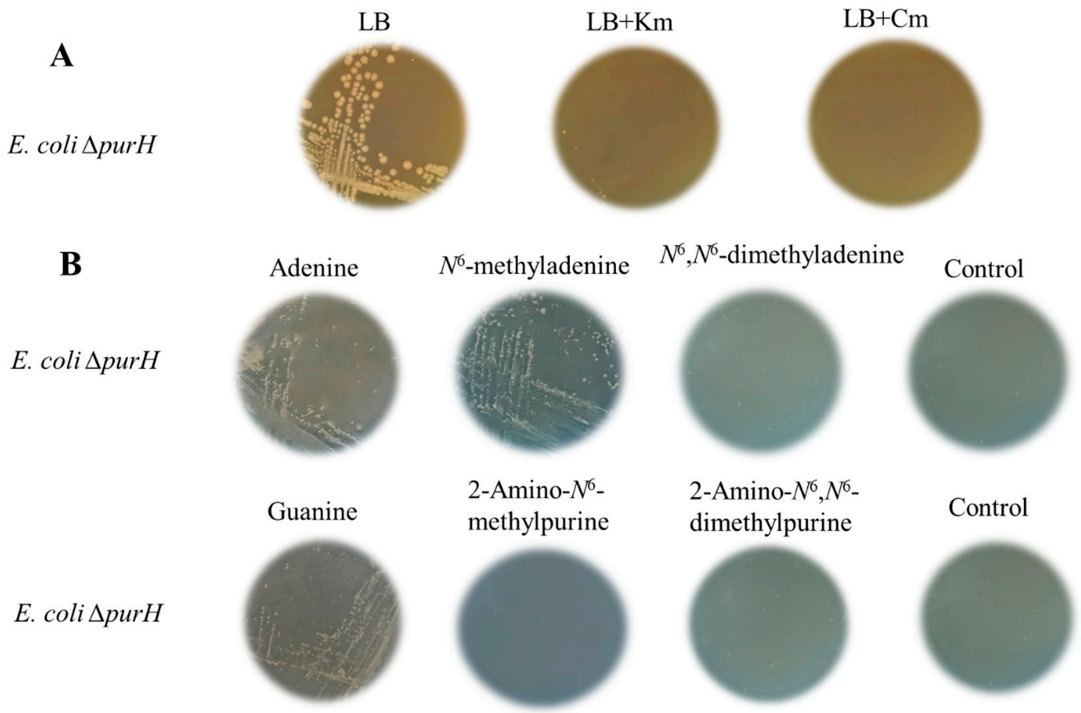

Figure S1. Growth of the *E. coli* Δ*purH* strain in the LB with antibiotics or M9 minimal media supplemented with different heterocyclic bases. (**A**) Δ*purH* strain grown in LB medium supplemented with kanamycin or chloramphenicol. (**B**) Δ*purH* strain grown in M9 agar minimal medium supplemented with adenine, N^6-methyladenine or N^6,N^6-dimethyladenine and also guanine, 2-amino-N^6-methylpurine or 2-amino-N^6,N^6-dimethylpurine. M9 minimal medium without any heterocyclic bases was used as control. LB agar plates were incubated at 37 °C overnight, whereas M9 agar plates - at 37 °C for 7 days.

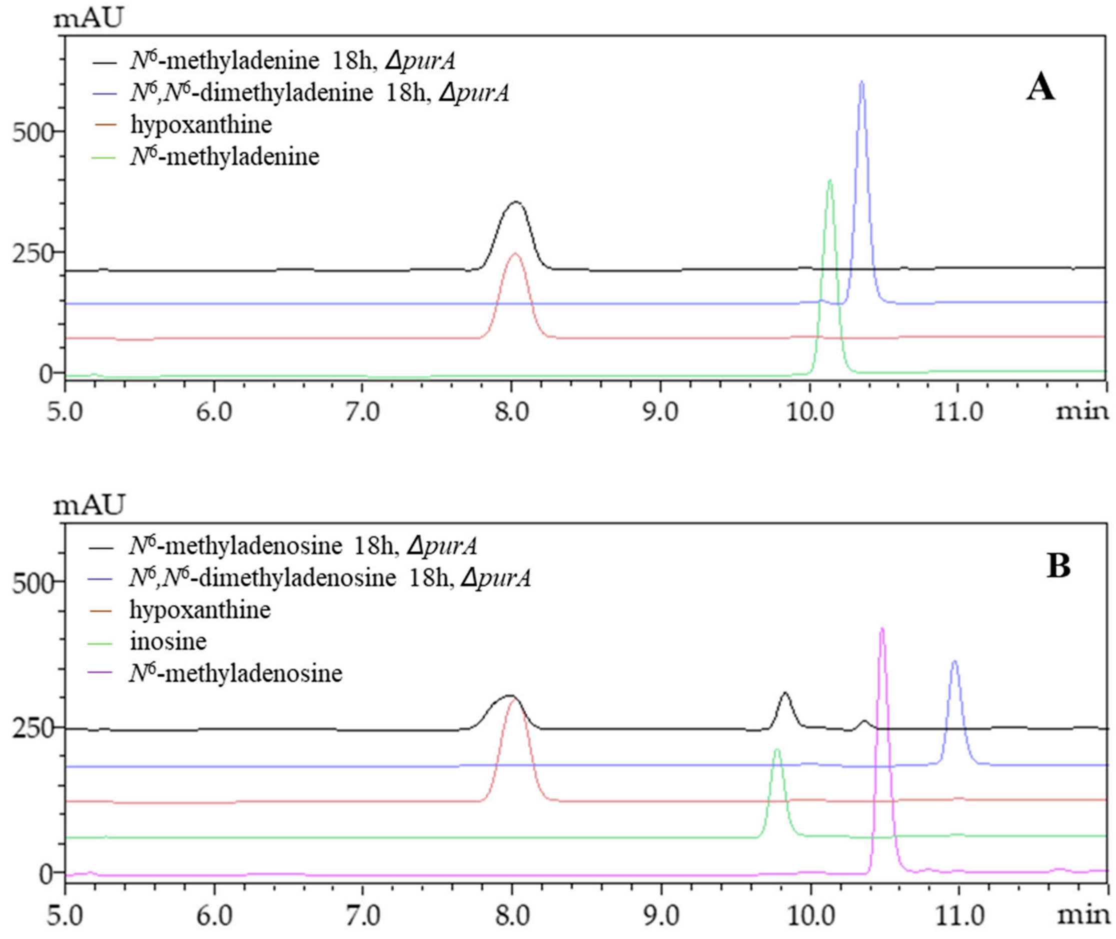

Figure S2. Accumulation of heterocyclic bases and nucleosides in medium supplemented with N^6-methylated adenines or adenosines. (**A**) accumulation of hypoxanthine in M9 minimal medium supplemented with N^6-methyladenine or N^6,N^6-dimethyladenine after transfer of the *ΔpurA*::Km mutant. (**B**) accumulation of hypoxhanthine and inosine in M9 minimal medium supplemented with N^6-methyladenosine or N^6,N^6-dimethyladenosine after transfer of the *ΔpurA*::Km mutant.
